# Notices, Reviews, &c.

**Published:** 1859-08

**Authors:** 


### NOTICES, REVIEWS, &c.

*Scientific American*, New York, $2 a year, is the only publication of its kind which has ever succeeded in this country, and that success is such that its publishers have enlarged and otherwise improved it, at a cost of eight thousand dollars, without any increase of the subscription price. It is considered authority on what pertains to science and mechanics, and well deserves it.

*Fruitless Preserves* are said to be made by boiling a pint of molasses about half an hour, then stir in rapidly three eggs, previously well beaten; boil a few minutes longer, and season with lemon or nutmeg.

*Blackwood's Magazine*, and the Four Quarterlies, written for by the first scholars of Great Britain, are reprinted by Leonard Scott & Co. 54 Gold Street, New York, at the low rate of $10 a year for the whole.

*The Scalpel*, for July, was not received, but is no doubt equal to any of its predecessors. $1 a year, quarterly, New York.

*Cool Nights* begin to come in August, when persons living in chill and fever localities should sleep with the outer windows and doors closed, and not expose themselves to the early morning air until breakfast has been taken.

